# Treatment of Infantile Paralysis of the Lower Limb
*Communicated to the Bath and Bristol Branch of the British Medical Association, November 28th, 1928.


**Published:** 1928

**Authors:** M. Forrester Brown

**Affiliations:** Surgeon to the Children's Orthopædic Hospital, Bath; etc.


### TREATMENT OF INFANTILE PARALYSIS OF THE LOWER LIMB.*

BY

M. Forrester Brown, M.S., M.D. Lond.,
*Surgeon to the Children's Orthopædic Hospital, Bath; etc.*

---

Infantile paralysis, or anterior poliomyelitis, furnishes a large proportion of the patients in the Bath, Somerset and Wilts Orthopædic Scheme, and the problems of treatment in cases where the lower limb is affected are both difficult and interesting.

*Examination of the patient.*—Before discussing the details of treatment it is necessary to say one word about diagnosis, for it is on an exact estimate of the relative loss of balance of the different parts of the limb that effective treatment is based.

Now, accurate diagnosis demands complete and careful examination, and I want, therefore, to plead for complete stripping of every case of infantile paralysis, even if the parent says it is only the great toe that is out of place ! I know the difficulties in private practice, where the practitioner who cannot "see what is the matter by the patient's face as she comes in at the door" is apt to be rejected as not knowing his job ; still, female limbs are not as elusive as they once were, and there are fashions in examination as well as in dress. If only the male practitioners would turn their attention to improving masculine attire, they might make their own work of examination much simpler. Even in our clinics the routine meets occasional obstacles, as when

* Communicated to the Bath and Bristol Branch of the British Medical Association, November 28th, 1928.

a parent informed the elderly V.A.D. that her shrieking son, aged three, was objecting to stripping "in front of all you girls—if you must know!"

Having got the patient stripped and one's attention directed simultaneously to all the joints of the lower limb, one has made a big step towards correct diagnosis.

Infantile paralysis is so essentially a disease with a scattered distribution, that it is rare to find the weakness, or at any rate the atrophy, restricted to one muscle, or even one group of muscles. On the other hand, it is nearly always of one region alone that the patient complains. Also one must remember that once treatment is started the patient becomes more attentive to his own symptoms and may discover the lesions in other areas, and if the medical practitioner has not pointed them out first, his splint or other treatment may be blamed for their appearance, *e.g.* a latent scoliosis may be progressive, and it not infrequently gets attributed to the leg-splint, though the latter may really be checking it.

The important steps in *diagnosis* are :—

1. To examine the patient from the front and see whether there is any lateral deviation from the normal axis of the limb at each level, *i.e.* outward from the mid-line — valgus — as in flat-foot, knock-knee, or shortening of the sartorius; or inward from it—varus —as in club-foot, genu varum, adducted hip.

2. To examine the patient from the side, to note anteroposterior deviations, *i.e.* reduction of the angle between leg and foot—calcaneus (long heel), increase of the angle between leg and foot; equinus (short tendo Achillis); recurvate knee, which may be secondary to equinus; flexed knee, which may be secondary to calcaneus; flexed hip with excessive forward tilting of pelvis and compensatory lordosis, etc.

3. Examination from behind to note unequal length of legs and corresponding tilting of pelvis. This tilt may be much diminished if the patient has the habit of bending the knee on the sound side, as some do.

4. After this the limb should be examined in recumbency with all tension off it. It saves much time and trouble to have a muscle chart on which to note down the response of each muscle in turn. The form designed by the Harvard Research Commission and adopted in modified form at the Bath Children's Orthopædic Hospital, is a very useful one, as it shows at the top the passive range of the joints from above downward and the muscles of the limbs also from above down, while the left and right are in the positions in which the observer would see them viewing a patient from the front, so that the table forms a rough sketch of the condition. Also measure for shortening of one lower limb.

In this connection one should call to mind a mechanical consideration that is apt to be forgotten in connection with the lower limbs, *i.e.* that in the normal functioning position of the limb, as in standing, or rising from a sitting position, the muscles act from below upwards, from their so-called insertions towards their so-called origins. Thus the foot muscles maintain the leg on the foot; the leg muscles straighten the thigh on the leg; the thigh muscles fix the pelvis on the femur; while the glutei and recti abdominis form a couple which reduce the forward tilt of the pelvis. The study of muscles on the recumbent subject in the dissecting-room is apt to give a false notion of muscles and the mechanics of the living body.

A second mechanical consideration of great importance is that in order to convert the jointed lower limb into a stable rod for the support of the body in the upright position, the antagonist muscles on opposite sides of each joint have to contract simultaneously, not alternately, as we are apt to think of their doing. Even in the act of walking, though the joint is moved into flexion by one set of muscles and the extensors lengthen correspondingly, they do not relax while any weight is on the limb, but maintain their postural tone constantly in health. It is the disturbance of this delicate co-ordination which makes many patients with poliomyelitis walk with a limp, apparently quite disproportionate to the muscle weakness that can be noted with the crude methods of examination at our disposal.

Thus, in the recumbent position a patient may seem able to abduct his hip against our resistance with great force, and we may be unable to detect much difference between the affected and unaffected sides. Put him on his feet and ask him then to stand on the sound limb only ; he maintains his body axis vertical and the pelvis horizontal by sheer force of the hip-abductors of the supporting limb, acting from it towards the trunk. Let him then stand on the weaker limb ; his muscles are unequal to supporting the trunk, and he reduces the stress by bringing the centre of gravity directly over the supporting limb, instead of hanging the trunk from it, as it were, *i.e.* his shoulders tilt over to the weak side. This test is a very delicate one. The phenomenon plays its part in Trendelenburg's sign for the diagnosis of congenital dislocation of the hip, in which the abductors are at a disadvantage from mechanical causes. Where dislocation complicates paralysis, as it may in anterior poliomyelitis, the sign will be present.

In examining muscles that are extremely weak, it is essential to test them in the neutral position as regards gravity, for many a muscle that will not attempt to lift the weight of the limb can be found to contract when that weight is removed. If it still fails to contract, it should be tested in a position in which gravity assists it, *e.g.* if the quadriceps tries to straighten the knee with the patient prone, gentle resistance by the examiner's finger may detect a tightening of the muscle. Such a degree of power is useless from the point of view of function of the limb, yet may mean that the muscle has the capacity to recover to a quite useful extent. Anyhow, it is alive, and no one can prophesy the possibilities; one can only assess its chance over a reasonably long period.

These briefly are the data on which treatment is based.

The *principles of treatment* are fourfold.

First, *rest* is helpful to the inflamed nerve cell, by cutting off as many incoming stimuli as possible, and to the paralysed muscle-fibre, cut off temporarily from its nutritional and stimulating centre, by preventing it from being over-lengthened; this rest to be continued during the whole of the acute stage. Unfortunately, the exact duration of the acute stage cannot be determined accurately, as it varies enormously in different cases; moreover, relapse appears to occur sometimes long after the initial attack. Many of the nerve-cells are only temporarily thrown out of action by vasomotor changes in their neighbourhood, and the process of resolution is bound to vary with the severity of these changes, as well as with other factors. It is to the restoration of these cells that treatment in the acute stage is directed, for those that are killed by the toxin cannot recover, and regeneration of nerve-cells does not occur.

Although all physicians have the same aim, their methods at this stage vary enormously, and with so erratic a disease it is difficult to appraise the value of any method. Still we should remember that, though it is impossible to cut off all afferent stimuli from a region of the spinal cord, and so rest a nerve cell entirely, yet every time we move a joint, which is supplied by that segment, or elongate a muscle, we are sending into it an avoidable stream of stimuli. When we consider this, we shall agree that it is wise to give complete recumbency to every case of infantile paralysis in the early stage, and to continue it till all signs of activity, such as muscle tenderness, have passed and spontaneous improvement is occurring in most or all of the weakened muscles.

The next step is *re-education* of muscles which have been paralysed or only disused. It is over this question of re-education that the greatest divergence of practice occurs. Some leave everything to nature, until perhaps gravity comes in to upset what was otherwise a balance among the recovering muscles and the child develops a clumsy habit of walking, or even contractures of certain joints. Others are so afraid that disuse will atrophy the muscles for ever, that they start very early whipping up the muscles into activity. If they are lucky, they take to themselves the credit of a fine recovery, forgetting that this disease gives spontaneously a good percentage of recovery, irrespective of any or every treatment. If they are unlucky they exhaust and paralyse for ever muscles which might have been saved. Lovett with his unique experience demonstrated this risk over and over again, and he recorded one case in his private practice, where a year after the acute attack a group of muscles was paralysed by over-exercise under skilled supervision.

In connection with re-education, it may be mentioned that *electrical treatment* is a useful adjunct for waking up what one may call lazy muscles, or perhaps more truly a lazy cerebral cortex, but it has never been proved to have the slightest direct favourable influence on the course of the disease itself; it may, however, easily become one of the agents through which the recovering nerve cells are stimulated to exhaustion. Therefore, if one is uncertain whether it is to be given by an expert—not an expert on batteries, but an expert on poliomyelitis!—it is best avoided. These matters of rest and re-education come usually into the sphere of the physician; but may an orthopædic surgeon plead that the physician, in dealing with the early stages, shall adopt the same mechanical principles which the orthopædic surgeon follows in the late stages? In a civilised country he ought not to be asked to deal with contractures of joints following poliomyelitis, because they should never have arisen.

The management of *contractures* represents the third phase in treatment, and it should consist almost entirely in preventing them.

They are due either to the action of gravity (or some mechanical force like heavy bed-clothes) causing over-stretch of paralysed, or weakened muscles, whose elasticity is temporarily in abeyance, or to the action of some unimpaired antagonist muscle doing the same. Such an over-stretch may be permanent, which is a calamity. More often it can be recovered from by relaxation of the muscle in its shortest position for weeks, or possibly for months on end. Such relaxation, followed by return of power in the muscle, explains most of the dramatic cures obtained by late orthopædic treatment. Much more valuable would be some undramatic care and patience in the earlier stages.

The prevention of over-stretching of one group and contractures of its opponents demands very careful and very regular supervision of the case from its earliest stages till full recovery develops. At first, if all the muscles seem equally affected, the anti-gravity ones should be given the benefit of splinting. Then if they begin to gain over their opponents, the balance must be changed, and re-adjustments made daily, or even twice a day.

When contractures have developed, they must be overcome very gradually, for the bones are brittle and more easily snapped than are the shortened ligaments ; also it is easy to produce a subluxation of the bones instead of a true correction, and this occurs readily if force is not used in the line of the deformity. For example, for a flexed knee the tibia must be pulled down from the femur without changing the angle, and not levered round the corner, which would only drive its head behind the femoral condyles, a secondary deformity very hard to reduce. Traction with adhesive strapping from the skin and some fixed splint, such as the Jones's hip abduction frame or the Thomas's knee splint, is the best for the hip and knee, because in these appliances side bandages can be used to steady the limb and prevent, or correct, lateral deviations of the limb, such as knock-knee or external rotation at the hip. The popular weight-and-pulley traction is very apt to allow these to develop, unless it is carefully controlled by troughs and gutters such as Rollier uses at Leysin. Moreover, in the acute stage the hip frame allows the muscles and joints to remain undisturbed during all nursing manipulations.

For the foot a series of plaster-of-Paris splints is generally best, as they cannot get out of place even with inefficient bandaging, as so frequently happens with wooden and other foot splints. The levers are so short and the joints so numerous in the foot that perfect position is difficult to attain in any other way. Sometimes in the acute stage there is a spasm of the tendo Achillis similar in type to that found with cerebral palsies, and almost as severe, and if this is neglected the resulting deformity is very hard to deal with; but if plaster is applied early, after a few weeks it can be removed and the contracture is found gone, while the muscle balance is likely to be the best for walking.

If contractures and over-stretch have been avoided in the way described during the recumbent stage of treatment, it only remains to determine what support the lower limb will require during standing and walking in order that they may not come on in the late stages from the persistence of the two causes noted above, *i.e.* gravity and unequal muscle balance, both of which will act with greater force in the functioning position of the lower limb. In other words, it is essential to provide for the stability of the limb that it may support the body weight. Herein lies the value of a thorough primary examination. If we have assessed correctly the degree of faulty balance and its level, the principles of the apparatus to be ordered will be simple.

Where the lateral deviation is a slight one and entirely postural, *i.e.* easily corrected by the examiner's hand, mere wedging up on one side of the sole and heel of the boot should suffice, provided that the correcting boot is worn during all weight-bearing. It is rarely wise to crook either sole or heel independently, as this puts a twist on the foot; occasionally it may be used to undo a mild twist, but it is much better to correct such a foot in plaster before trying adjustments to the boot. It must be borne in mind that it is only a boot or shoe with a firm upper, fastened on securely with laces that will hold an unbalanced foot on the tilted sole; a slipper or sandal will allow return of the deformity by permitting the foot to slide across the lax upper. This point is often overlooked, especially by parents, who must be warned of it.

When the deviation is too severe for a tilted boot to correct it, if it affects the foot only, it can be dealt with by a T-strap sewn along the edge of the boot on the side towards which one desires to pull (outside for varus and inside for valgus deformities); the strap is then buckled round a light iron on the opposite side of the limb.

If the knee takes part in the deformity, such a short iron would throw undue strain on its muscles and ligaments, so that a Thomas's walking caliper should be substituted for it, as the T-strap can equally well be buckled to that, while a special bandage or strap can be put on to draw the knee to the required side.

If the hip is unstable, a well-fitting caliper with its ring taking weight from the tuber ischii will probably provide sufficient stability for the limb, but such a splint will do nothing to overcome a fixed lateral deviation, which must be corrected either by a plaster spica, applied from the ribs to the toes (as rotation is always associated with the other deformities), or else by recumbency on a Jones's frame. This applies equally to abduction contractures brought about by shortening of a hypertrophied sartorius, and adduction contractures, both types being usually accompanied by flexion.

If the abductors are absolutely paralysed in a heavy subject, it is occasionally necessary to supply a short spinal brace and attach the caliper to it by hinges which must act in two directions, *i.e.* abduction and also flexion. If they only hinge forward and backward for walking and sitting, and do not give at all sideways, the lateral strain on them will be so enormous that the bars will continually be breaking. It is rarely, if ever, desirable to fix one hip completely by a leg extension from the spinal jacket, for such a method of walking is difficult to anyone, let alone a patient who is sure to have some general weakness in addition. It is useless to attach the hinge to a slack pelvic bar, as instrument-makers so often do. If the patient has strong trunk muscles, he can usually manage in a caliper alone. If his trunk is weak, he requires the pelvis to be steadied on the spine by a well-fitting jacket, not an uncomfortable bar that keeps slipping.

As regards the introduction of hinges into such leg and knee-irons, it may be said at once that they add greatly to the weight of the apparatus, as well as to its expense, and they demand very accurate fitting, as well as elaborate adjustments for growth in a child. Thus for a child a knee-hinge is scarcely ever worth while, and the ankle-hinge is well substituted by a round end to the iron, playing in a tube in the heel of the boot. For an adult who has to travel regularly in public conveyances and sit at work, a hinge for the knee, which can be locked on standing, should be fitted to the caliper. It should act slightly below the joint level, never above that.

As to antero-posterior deflections, equinus with shortening of the tendo Achillis should be first overcome by a series of plasters, best applied with loose wool below the heel, which sinks as the patient walks, while a metatarsal bar of plaster prevents softening of the cast in front. It is wonderful what tight heels can be stretched by this method, so that it is hardly ever necessary to lengthen a tendo Achillis by operation. When the deformity has been corrected, any remaining foot-drop can be dealt with by a side iron and an ankle-stop to check ankle movement downward. As foot-drop is generally due to weakness of the tibialis anticus, an invertor muscle, it is best to put the iron on the outer side of the limb and apply an inside T-strap.

Calcaneus, a long heel, from paralysis of the calf muscles, is best treated in its milder grades by raising the heel of the boot, or one may apply an iron with stop to check dorsiflexion. If severe, it can only be well corrected by a bone operation.

Flexion of the knee nearly always accompanies calcaneus. Raising the heel will correct it. If due to complete paralysis of the quadriceps, it may demand the support of a caliper, for unless the alinement of the foot is perfect, the knee is very apt to give sideways into genu valgum, and often gets a twist of the tibia outward on the femur, much easier to prevent than to cure.

Recurvate knee is associated with paralysis of the hamstrings, but may be induced or aggravated by equinus. If severe it requires a caliper with a firm sling behind the knee. If the hamstrings are good, but the quadriceps weak, this tendency of equinus to keep the knee recurvate can be used to prevent the knee giving way in flexion, since if the heel of the boot is kept raised, the patient can usually control the knee without any knee splint ; but this is unsafe in a case of flail knee.

As already noted, a flexed hip must be corrected in recumbency and then supported by a caliper. It is not practicable to keep the hip constantly extended once the patient is up and about, owing to the discomfort of sitting.

As regards *stabilisation by operation*, many methods have been devised for the lower limb, but only a few are satisfactory. If more than one small muscle or part of an important one is gone from the lower limb, no re-arrangement of tendons by a transplant operation can possibly restore the balance so perfectly as to counteract the deforming force of the body-weight. Therefore a transplant must either be done as a temporary measure, because the child's foot is too small for a bone-fusion, or the patient must be content to wear irons for years.

In the knee a fusion operation is rarely needed and makes the limb very cumbersome. In the hip it is rarely needed except after occurrence of a dislocation, and then it is most difficult to ensure firm ankylosis.

The foot, being at the end of the rod, endures most leverage, and has a complicated mechanism of little joints playing in various directions, which are very difficult to control when any muscles are missing, or even are exhausted. In the foot stabilisation by arthrodesis is most often called for and gives the most satisfactory results. Naturally anyone would prefer the springy mechanism of the normal foot to the inelasticity of a fused one, but in severe cases of anterior poliomyelitis there is no hope of enjoying such a springy foot. There is only a choice between a stable base to the limb in good position, which avoids strain on the links above ; and an unstable affair with steadily increasing deformity, which slowly but surely involves in its own defect the joints above it, so that the strain may ultimately be felt right up the spine. To this kind of foot fusion of the tarsal joints by such methods as Dunn's and Hoke's offers a satisfactory result, provided great care is taken in setting the foot correctly on the leg, more especially as regards the neutral position of the subastragaloid joint, when the final plaster is applied. Also the foot must not be walked on for many weeks even in plaster, or the soft callus may become deformed ; the plaster should be kept on for six months, followed by an iron for another six months. Once the fused bones are set hard, there is no risk of recurrence of deformity.

As regards compensating for shortness of a lower limb, in a growing child compensation should be complete to within half an inch to avoid scoliosis. With raises over one inch and a half the heel should be kept half an inch higher than the sole. In adults the lowest comfortable raise should be chosen.

